# Intervening factors in the feeding of infants vertically-exposed to HIV: an integrative review

**DOI:** 10.26633/RPSP.2017.114

**Published:** 2017-11-30

**Authors:** MarÍlia Alessandra Bick, Polyana de Lima Ribeiro, Tamiris Ferreira, Stela Maris de Mello Padoin, Cristiane Cardoso de Paula

**Affiliations:** 1 Universidade Federal de Santa Maria Universidade Federal de Santa Maria Santa Maria Brazil Universidade Federal de Santa Maria, Santa Maria, Brazil.

**Keywords:** HIV, acquired immunodeficiency syndrome, infectious disease transmission, vertical, infant nutrition, infant formula, review, VIH, síndrome de inmunodeficiencia adquirida, transmisión vertical de enfermedad infecciosa, nutrición del lactante, fórmulas infantiles, revisión, HIV, síndrome de imunodeficiência adquirida, transmissão vertical de doença infecciosa, nutrição do lactente, fórmulas infantis, revisão

## Abstract

**Objective:**

*To evaluate the available scientific literature on factors that may intervene in the adequate feeding of infants vertically-exposed to HIV*.

**Methods:**

*This was an integrative review of the literature, performed on the LILACS, PubMed and Scopus databases in February 2017. The search was guided by the question “What are the factors involved in feeding infants vertically-exposed to HIV.” Selected studies met the inclusion criteria of being research articles published in English, Portuguese, or Spanish. Articles excluded were those on exclusive breastfeeding. There was no need to perform a temporal cut off of the studies*.

**Results:**

*In all, the 32 primary studies selected showed that intervening factors were of three types: individual, such as maternal feelings and desires, beliefs, and practical difficulties; social, such as socioeconomic conditions, social support, and stigma; and political, such as health services structure and organization, supplies, health care guidance, and the knowledge and attitudes of health care professionals*.

**Conclusions:**

*The factors that interfere with feeding infants vertically-exposed to HIV may be independent or associated with each other. To reduce the risk of inadequate nutrition and its associated diseases, actions must be taken to identify and minimize these factors, guaranteeing a better quality of life and reduction of infant morbidity and mortality*.

## RESUMEN

**Objetivo:**

Evaluar la bibliografía científica disponible sobre los factores que pueden influir en la alimentación adecuada de lactantes expuestos a la transmisión vertical del VIH.

**Métodos:**

En febrero del 2017, se realizó una revisión integradora de la bibliografía en las bases de datos LILACS, PubMed y Scopus. La búsqueda fue orientada por la pregunta "¿cuáles son los factores involucrados en la alimentación de los lactantes expuestos a la transmisión vertical del VIH?". Los estudios seleccionados cumplían el criterio de inclusión de ser artículos de investigación publicados en inglés, portugués o español. Se excluyeron los artículos sobre la lactancia materna exclusiva. No fue necesario establecer un límite temporal para los estudios.

**Resultados:**

En total, los 32 estudios primarios seleccionados mostraron que intervinieron factores de tres tipos: individuales, como los sentimientos y deseos maternos, las creencias y las dificultades prácticas; sociales, como las condiciones socioeconómicas, el apoyo social y el estigma; y políticos, como la estructura y organización de los servicios de salud, los suministros, la orientación sobre la atención de salud y los conocimientos y actitudes de los profesionales de la salud.

**Conclusiones:**

Los factores que interfieren en la nutrición de los lactantes expuestos a la transmisión vertical del VIH pueden ser independiente o estar asociados entre sí. A fin de reducir el riesgo de una nutrición inadecuada y las enfermedades asociadas con ella, se deben tomar medidas para establecer y reducir al mínimo esos factores, garantizar una mejor calidad de vida y reducir la morbilidad y la mortalidad de los lactantes.

## RESUMO

**Objetivo:**

Avaliar a literatura científica existente quanto aos fatores que podem interferir na alimentação adequada de lactentes expostos à transmissão vertical do HIV.

**Métodos:**

Estudo de revisão integrativa da literatura conduzido nas bases de dados LILACS, PubMed e Scopus em fevereiro de 2017. A busca foi norteada pela seguinte pergunta: “Quais são os fatores intervenientes na alimentação de lactentes expostos à transmissão vertical do HIV?”. Os estudos selecionados satisfizeram os critérios de inclusão, a saber: artigos de pesquisa publicados em inglês, português ou espanhol. Foram excluÍdos os artigos sobre aleitamento materno exclusivo. Não foi necessário fazer um recorte temporal dos estudos.

**Resultados:**

Os 32 estudos originais selecionados revelaram três tipos de fatores intervenientes: individuais, como sentimentos e desejos, crenças e dificuldades práticas das mães; sociais, como nÍvel socioeconômico, apoio social e estigma, e polÍticos, como estrutura e organização dos serviços de saúde, provisões, orientação de cuidados da saúde e conhecimento e atitudes dos profissionais da saúde.

**Conclusões:**

Os fatores que interferem na alimentação dos lactentes expostos à transmissão vertical do HIV podem ocorrer independentes ou associados entre si. A fim de reduzir o risco de nutrição inadequada e doenças associadas, devem ser tomadas medidas para identificar e minimizar esses fatores, assegurando melhor qualidade de vida e redução da morbidade e mortalidade infantis.

The global epidemic of human immunodeficiency virus (HIV) has evolved over time—the highest rates of new cases are now among women of childbearing age, thereby increasing the number of infants at risk of infection by vertical transmission (1). In 2015, there were approximately 150 000 new HIV cases among infants worldwide (2).

HIV vertical transmission can happen at three points: during pregnancy, during labor and childbirth, and during each breastfeeding session (2). In order to ensure the protection of life and to reduce the chances of vertical transmission (3), timely access to prophylactic and antiretroviral treatment is imperative (4).

According to the World Health Organization (WHO), breastfeeding accounts for 30%–50% of HIV vertical transmission when prophylactic measures and treatment are not adhered to; breastfeeding also reduces the positive impact of preventive interventions during pregnancy and parturition (4). While breastfeeding carries the risk of HIV transmission, alternatives have significant health risks for the infant, including malnutrition, morbidity, and mortality (5).

The most appropriate feeding choice depends on various factors, such as the individual mother’s health status and socioeconomic conditions and the availability of health services that offer counseling and support. For HIV positive mothers, the WHO recommends breastfeeding only until 6 months of age; however, if the replacement food meets the criteria of being acceptable, viable, accessible, sustainable, and safe, the exclusion of breastfeeding is recommended (4). This guideline is justified by the increased risk of morbidity and mortality due to malnutrition and infectious diseases that can result when breastmilk substitutes lack sufficient nutrients and/or when basic sanitation and safe food and water are not available (5).

Post-partum prevention of vertical transmission becomes the responsibility of the caregiver, whether a blood relative or not. When wholly supported and guided by health professionals, the caregiver can be empowered to improve the child’s quality of life (3). However, when guidance and support are insufficient or inadequate, the infant may have unmet nutritional needs, as reported by Freitas and colleagues (6) who found that information on appropriate feeding of this at-risk group was lacking.

The effectiveness of any recommendations is directly related to the psychosocial, cultural, and biological specifics of each family; and to wholly comprehend the reality of each, it is imperative that health professionals take an approach that fosters a relationship of caring and mutual respect (7).

Given that there is wide variation in the factors that both positively and negatively influence the food choices made for infants vertically-exposed to HIV, the objective of this study was to evaluate the available evidence on intervening factors when breastfeeding was not exclusive.

## MATERIALS AND METHODS

This was an integrative review (8) that aimed to gather and summarize research results to answer the question, “What are the social, political, and individual inter-vening factors in the feeding of infants vertically-exposed to HIV who were not exclusively breastfed?”

A bibliographic search was conducted in February 2017 of the Latin American and Caribbean Health Science Literature (LILACS), the United States National Library of Medicine (PubMed), and Elsevier SciVerse Scopus (Scopus) databases. The following keywords were used on PubMed and Scopus: “HIV or acquired immunodeficiency syndrome” and “infant nutritional physiological phenomena or feeding practices or bottle feeding or infant formula.” The following keywords were used on LILACS: “HIV or AIDS or *Sindrome da imunodeficiência adquirida* or *vírus da imunodeficiência humana* or *Soropositividade para HIV”* and *“neonato* or *criança* or *pediatria* or *materno-infantil* or *materno-fetal”* and *“nutrição* or *alimentação* or *alimentação artificial* or *cuidadores* or *cuidado* or *cuidado infantil.”*

Initially, the search resulted in 2 827 papers. For the feasibility of the analysis, in PubMed and Scopus, the following filters were applied: article type (clinical trial, comparative study, controlled clinical trial, multicenter study, observational study, controlled randomized clinical trial); species: human; language: English, Portuguese, or Spanish; ages: infant (birth - 23 months) and child (birth - 18 years). The total search results were 531 publications.

The following selection process was followed: establishing the integrative review’s objective, applying inclusion and exclusion criteria, defining the information to be extracted from the selected articles, analysis of results, discussion of results, and presentation (8). For this review, articles that met the inclusion criteria were research papers published in peer-reviewed journals, in Portuguese, English, or Spanish. Articles excluded were those whose study population was exclusively breastfed. There was no need to perform a temporal cut off the studies.

**FIGURE 1.**
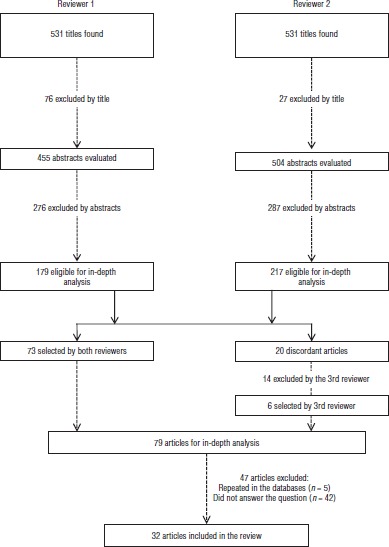
Flowchart of selection process for a review of the literature on “Intervening factors in the feeding of infants vertically-exposed to HIV” available from LILACS, PubMed, and Scopus, 2017

***Source:*** Prepared by the authors from these study data.

To minimize error or omission, each of two researchers independently reviewed the 531 possible studies. According to the titles, abstracts, and lastly, the entire text, those that did not respond to the research question and objectives were eliminated. A third researcher read any articles where there was a discrepancy between the first and second researcher’s recommendation (Figure 1). Critical evaluation was performed in accordance with the Melnyk and Fineout-Overholt’s hierarchy of evidence levels (9) through the analysis of the type of clinical question and of each study’s methodology.

The classification was carried out according to whether the study was (a) directed at treatment or intervention, (b) directed at prognosis or etiology, or (c) directed at understanding emotional or qualitative aspects.

There were 32 publications that addressed the research question and review objective. Each of the 32 studies was recorded in an instrument that collected bibliographic information, country(ies) of focus, knowledge subarea, objectives, methodology, results, and level of evidence.

## RESULTS

Regarding the characteristics of the analyzed articles (*n* = 32), there was a predominance of studies conducted in Africa (72%; *n* = 23) probably due to the large number of HIV cases among the population. In the knowledge area, there was a concentration of papers by multiprofessional teams (57%; *n* = 18). The temporal distribution showed that 50% were published in 2003 - 2007 (*n* = 16). This shows that research during the most recent decade has be waning at a time when the need for information on safe feeding among the study population has grown (Table 1).

**FIGURE 2.**
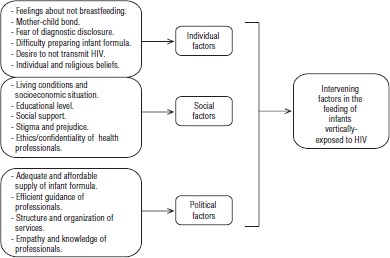
Schematic illustration of the intervening factors in the feeding of infants vertically-exposed to HIV, as evidence by the literature available from LILACS, PubMed, and Scopus, 2017

***Source:*** Prepared by the authors from the study process.

Analysis of the 32 publications enabled identification of intervening factors in the feeding of infants vertically-exposed to HIV (Figure 2). Results of these studies contemplated three interdependent groups of factors— individual, social, and political. The individual factors refers to biological, emotional, cognitive, attitudinal, and social relationships; the social, to cultural, community, and economic aspects that determine access to goods and services; and the political, to social resources that protect a citizen’s physical, mental and social well-being. Further details on the different individual and collective situations are available in a Supplementary File.

## DISCUSSION

### Individual factors

The individual factor group examined behaviors that intervened positively or negatively with feeding of infants vertically-exposed to HIV. These were individual behaviors that point to the need for understanding each particular situation and the specifics for each woman, against a backdrop of cultural heterogeneity.

The studies indicated that depriving an infant of breast milk was a trigger for feelings of guilt, pain, and sorrow (10-14). Reasons cited were a mother’s inherent desire to breastfeed, family and social pressure, cultural and religious issues, including rejection of a mother who does not breastfeed and withdrawal of family support (11-25). Out of fear, some use rea-sons deemed more socially acceptable to justify their decision to not breastfeed—e.g., insufficient breast milk, anemia, or using antibiotics (10, 12-17, 22, 23, 26-29). However, the desire to protect the child from HIV, according to these studies, was a factor that facilitated the acceptance of replacement of breastfeeding (13-15, 17, 20, 21, 25, 27-31). Related to this, maintaining and strengthening the mother-child bond can be fostered by other forms of physical contact, often provided with greater care and attention (11, 15, 32).

**TABLE 1:** Characteristics of articles analyzed for a review of the literature on “Intervening factors in the feeding of infants vertically-exposed to HIV” available from LILACS, PubMed, and Scopus, 2017

Characteristics	n	%
Geographic area	
Africa	23	72
Brazil	8	25
Colombia	1	3
Knowledge areas	
Multi-professional	18	57
Nursing	6	19
Medicine	6	19
Nutrition	2	5
Year of publication	
2003-2007	16	50
2008-2012	10	32
2013-2016	6	18

***Source:*** Prepared by the authors from the study data.

The studies also indicated that the success of strategies for avoiding vertical transmission depended the mother’s understanding of the importance of not breastfeeding and the effects of HIV transmission (13, 15, 19, 25, 28, 30-34). Mothers who adhered to antiretroviral treatment and who were receiving follow-up health care were more likely to comply with the infant feeding recommendations and more likely to prevent vertical transmission (25).

### Social factors

The social factor group analyzed access to information, health services, and health and social welfare conditions that intervene in the feeding of infants verticallyexposed to HIV. The studies indicated that these aspects were reflected in the family’s socioeconomic conditions and by the infrastructure of the community where the infant resides (10-12, 15-17, 22-25, 27, 30, 31, 35-38). Availability of piped, potable water and electricity was pointed out as a factor that facilitated feeding by infant formula, due to the need for clean water and refrigeration (16-19, 21, 22, 27, 29, 30, 36-38). A low level of education was found by most studies to affect proper formula preparation, as well as to impede the mother’s understanding of how replacement of breastfeeding can avoid vertical transmission of HIV (10, 14, 15, 19, 28, 30, 31, 35-37). Studies also suggested that the higher the level of education and family socioeconomic status, the more likely the mother was to follow health guidelines and introduce solid food after 6 month of age (24, 25, 33).

It is notable that financial difficulties and social precariousness may favor inadequate feeding (10, 11, 14, 17, 25, 26, 33, 37, 39, 40). The articles reviewed reveal that the practice of excessive dilution of formula is frequent, often attributed to food insecurity and the need to share any food with siblings (14, 18, 25, 36, 38-40). In addition, because families have difficulty paying for infant formula, introduction of solid food becomes necessary earlier than recommended. The foods offered are rich in carbohydrates and fats and deficient in proteins, vitamins, and minerals— extremely necessary at this age—and contribute to poor growth and development (14, 17, 18, 25, 26, 39, 40). Studies conducted in Brazil and in Africa show that when infant formula cannot be purchased or otherwise obtained, family/solid food often starts before 2 months of age (10, 17, 26, 32, 33, 40).

The studies demonstrated that women who opt to substitute with milk formula suffer prejudice and are stigmatized as HIV carriers by the community (13-15, 17, 19, 20, 24, 26, 28, 37), in addition to experiencing pressure, harassment, isolation, and/or violence from the family (17, 20, 28, 29, 37).

The proximity of health care services where antiretroviral medications and infant formula are provided was mentioned as a complicating factor because of the stigma by the community and the fear of the mother’s HIV status being discovered (14, 40). However, many mothers stated that when they revealed their HIV diagnosis to friends or family, they continued to receive support, whether emotional or logistical (10, 14, 17, 24, 25, 31, 37). Revealing the diagnosis to the partner/spouse influenced feeding choices, especially when the couple established strategies to deal with their reasons for not breastfeeding (13, 14, 24, 25, 28, 29, 31, 37).

The studies also showed that health professionals still miss the mark with regard to ethics, by discriminating or not respecting the confidentiality (11, 15, 40). Health professionals must try to understand each woman’s needs, and do their utmost to help each mother feel safe and empowered to care for her infant in a, risk-free and nutritionally appropriate way. Thus, with the professional’s support, it is possible to keep the HIV status confidential and allow the healthy development of any child in a family living with HIV (41).

### Political factors

The political factor group was configured to gather information on health and educational resources and investments that may intervene in the feeding of infants vertically-exposed to HIV. In this sense, an absence of receptivity was evidenced, and these factors had an influence on the lack of understanding between health professionals and the HIV-positive mothers (11, 15, 29, 36, 37, 40). This particular relationship requires acceptance, empathy, respect, cordiality, and patience, so that the health guidelines are effectively adopted (11, 15, 19, 20, 29, 30, 40). Proper counseling and support can empower mothers, turning recommendations into actions through their decisive and conscious attitudes toward the health of their children, including adequate nutrition (24, 39, 41).

There was also evidence for the need to improve the knowledge of health profes-sionals regarding guidelines for feeding children vertically-exposed to HIV. One study showed that only 14% of professionals felt their knowledge on the subject was adequate (41): they were aware that formula feeding increases the risk of infant morbidity and mortality; however, the guidance on safe nutrition for those exposed to HIV is at odds with WHO recommendations (25, 41).

Most of the studies reviewed pointed to a need for restructuring and reorganizing health services to guarantee the supply of necessary aids for improving the quality of life and health of HIVpositive mothers and their children (11, 15, 19, 23, 30, 39, 40).

A number of studies revealed that, in some places, infant formula is distributed free of charge to mothers with HIV (10, 17-19, 22, 26, 27, 29, 31, 32, 34, 35, 39, 40). However, findings also found that there are flaws in distribution, mostly shortages, that is, quantities insufficient to provide infant formula up to 6 months of age (13, 16-19, 22, 27, 35, 40). Furthermore, there are too often bureaucratic issues that disrupt the availability of the donated infant formula (13, 14, 16-19, 22, 31, 35, 40).

### Limitations

Most studies on the feeding of HIV- exposed infants have been conducted in Africa; therefore, a lack of studies in countries with distinct socioeconomic and cultural conditions is a limitation of this study.

### Conclusions

This review of the literature highlights the interdependence among individual, social, and political factors and their influence on the feeding of infants vertically-exposed to HIV. Training and continuing education for health professionals is needed, as shown by the difficulties reported with attending to families living with HIV. A better understanding of the reality in which these families live is needed in order to improve the effectiveness of health professionals. HIV-positive mothers need to be empowered to provide adequate, safe, accessible, and affordable feeding of their infants, and family support was shown to be imperative. All these intervening factors must be addressed to prevent HIV transmission, reduce the risk of malnutrition and associated diseases, and ensure a better quality of life for infants vertically-exposed to HIV.

### Acknowledgements

The authors wish to thank the reviewers for their input on an early version of the manuscript.

### Funding

Study funding was received under the Edital Universal 01/2016 (faixa A) of the National Council on Scientific and Technological Development (CNPq) of the Ministry of Science, Technology, Innovation and Communications of Brazil.

### Disclaimer

Authors hold sole responsibility for the views expressed in the manuscript, which may not necessarily reflect the opinion or policy of the *RPSP/PAJPH* and/or PAHO.
